# Relieving or aggravating the burden: Non-communicable diseases of dual users of electronic and conventional cigarette in Indonesia

**DOI:** 10.18332/tid/175755

**Published:** 2024-01-10

**Authors:** Faizal R. Moeis, Risky K. Hartono, Renny Nurhasana, Aryana Satrya, Teguh Dartanto

**Affiliations:** 1 Institute for Economic and Social Research, Faculty of Economics and Business, Universitas Indonesia, Jakarta, Indonesia; 2 Department of Economics, New York University, New York City, USA; 3 Center for Social Security Studies (CSSS-UI), Universitas Indonesia, Jakarta, Indonesia; 4 Universitas Indonesia Maju, Jakarta, Indonesia; 5 Urban Studies Program, School of Strategic and Global Studies, Universitas Indonesia, Jakarta, Indonesia; 6 Department of Management, Faculty of Economics and Business, Universitas Indonesia, Jakarta, Indonesia; 7 Department of Economics, Faculty of Economics and Business, Universitas Indonesia, Jakarta, Indonesia

**Keywords:** disease, dual user, e-cigarette, Indonesia, smoking

## Abstract

**INTRODUCTION:**

Conventional (tobacco) and e-cigarette smoking prevalence is a growing concern in Indonesia. It has worsened as e-cigarettes complement conventional cigarettes, resulting in dual users, potentially causing an additional burden in terms of health.

**METHODS:**

Our study is a secondary data analysis of the 2018 National Basic Health (Riskesdas) Survey. The sample is limited to respondents aged 15–64 years who either only used e-cigarettes (e-cigarette single users), only used conventional cigarettes (conventional cigarette single users) or used both e-cigarettes and conventional cigarettes (dual users) in the last month. The sample size of the data was 174917 individuals. Our analysis utilized the logit and negative binomial regression to test whether the type of smoking behavior was associated with reporting to have a non-communicable diseases (NCDs) and multimorbidity, respectively.

**RESULTS:**

We found that: 1) dual users are positively associated to report having NCDs, such as liver failure (AOR=2.38; 95% CI: 2.32–2.44), diabetes (AOR=1.53; 95% CI: 1.50–1.57), hypertension (AOR=1.49; 95% CI: 1.48–1.51), and gum diseases (AOR=1.74; 95% CI: 1.73–1.74) compared to single users; and 2) e-cigarette single users are positively associated with reporting to have NCDs such as asthma (AOR=3.11; 95% CI: 3.01–3.22) and diabetes (AOR=16.01; 95% CI: 14.57–17.59), and dental problems such as broken teeth (AOR=1.04; 95% CI: 1.03–1.06), and they have disease multimorbidity compared to conventional cigarette single users.

**CONCLUSIONS:**

Simultaneous control of the consumption of e-cigarettes and conventional cigarettes is essential. In addition, it is important to promote policies to increase the price of e-cigarettes and conventional cigarettes to reduce smoking prevalence and prevent dual users. Moreover, as there are negative health consequences for conventional and e-cigarette single users or dual users, the most effective alternative is to stop smoking, not switching products.

## INTRODUCTION

Indonesia has one of the highest smoking rates in Asia^1^. Recently, Indonesia has also faced the emergence of electronic cigarettes (e-cigarettes). Initially, the prevalence of e-cigarette smoking in people aged ≥15 years was at 0.3% in 2011^2^. However, the National Socioeconomic Survey data showed that e-cigarette user prevalence increased by sevenfold in 6 years, reaching 2.3% in 2017 and only slightly decreased to 2.1% in 2019. Moreover, the increased prominence of e-cigarettes is heavily concentrated in youth, who integrated e-cigarettes into their lifestyle^3^. This is in line with the Indonesian 2018 National Basic Health Research (Riskesdas) where the percentage of the population who had used e-cigarettes was the highest in the those aged 10–14 years (10.6%) and 15–19 years (10.5%)^4^.

The use of e-cigarettes was initially expected to replace conventional cigarettes^5^. However, e-cigarettes have appeared to become complements of conventional cigarettes, resulting in the emergence of dual users^6^. In this study, the term ‘Dual users’ refers to people who were both conventional smokers and e-cigarette users during the month before the survey^7^. It is similar to the data found in Indonesia; active e-cigarette users are predominantly also conventional active smokers. Between 2017 and 2019, the percentage of dual users in Indonesia from e-cigarette users was (96–97%), which was higher compared to other countries, such as South Korea (85%)^8^. As the products of conventional cigarettes and e-cigarettes are used complementarily, e-cigarettes may cause an additional burden to the users compared to those who only use conventional cigarettes^8,9^.

Indonesia has implemented several fiscal policies to control e-cigarette usage. In 2018, e-cigarette liquid was given a 57% tax on its retail price, exceeding the average tax on conventional cigarettes. Moreover, in 2022, e-cigarettes were required to provide detailed information about the product, including contents, the address of the manufacturer or importer, warnings, and health information. However, there are not any non-fiscal regulations that limit the usage of e-cigarettes at the national level, making Indonesia’s control on e-cigarettes weak^10^. Without additional policies to control the e-cigarette phenomenon, the damage caused by dual users and e-cigarette usage threatens the third goal (Good Health and Well-Being) of the Sustainable Development Goals and Indonesia’s vision for ‘Better Human Resources for an Advanced Indonesia’.

The analysis of dual and e-cigarette usage on health in Indonesia remains limited. Most studies in Indonesia have focused on factors associated with e-cigarette usage^11^ and the perception of e-cigarette usage^12^. Furthermore, the health impact of e-cigarettes has been studied in other countries, albeit with differing degrees of severity compared to conventional cigarettes^13,14^. Thus, our study had two aims: 1) to assess the association between dual use and non-communicable diseases (NCDs) compared to single-use; and 2) to assess the association between single e-cigarette use and NCDs, compared to single cigarette use.

## METHODS

### Data sources and tools

This study is a secondary data analysis of the National Basic Health (Riskesdas) dataset, comprising cross-sectional observations. Riskesdas is a survey conducted by Indonesia’s Ministry of Health every five years. It is used to analyze the prevalence of health issues (e.g. stunting, smoking, alcohol usage, etc.) and diseases (e.g. NCDs and infectious diseases) in Indonesia. The Riskesdas data collection has gone through ethical clearance and the present study was also permitted by Indonesia’s Ministry of Health to utilize the data. The study focused on respondents aged 15–64 years (productive age) and either used e-cigarettes or conventional cigarettes in the last month. The sample size of the 2018 Riskesdas in the study included included 174917 individuals. After utilizing the individual frequency weights, the Riskesdas samples represented 58342892 individuals.

### Measures

The diseases analyzed in the study are NCDs, including asthma, hypertension, stroke, liver failure, rheumatism, diabetes, heart disease, broken teeth, mouth ulcers, and gum diseases. In general, the survey asks the respondents whether they have ever been diagnosed by a doctor for a specific disease. The study also provides an analysis regarding the multimorbidity of the respondents, defined as the total number of NCDs reported by the respondent. The diseases included in multimorbidity include asthma, hypertension, stroke, liver failure, rheumatism, diabetes, and heart disease. Smokers were defined as people who had smoked in the last month. Conventional smokers included smokers who smoked clove cigarettes, white cigarettes, rolled cigarettes, or shisha in the last month. At the same time, e-cigarette smokers included smokers who smoked electronic cigarettes in the last month.

### Statistical analysis

In the first step of the analysis, we compared dual users with single users (electronic or conventional cigarette single users). We compared single e-cigarette users and conventional cigarette single users in the second step, while dual users were excluded from the analysis. The study utilizes Stata 16 software for the data analysis. The study employs two logit regressions to analyze the association between dual users and single e-cigarette users with reported NCDs. This choice was determined by the nature of the dependent variable (a binary variable, whether an individual reported having the NCD or not). Probability is described through the adjusted odd ratio (AOR). The primary independent variable in the first model is a dummy variable, whether the individual is an active user of both conventional cigarettes and e-cigarettes (dual user) or only an active user of e-cigarettes/only an active user of conventional cigarettes (single user). Whereas the main independent variable in the second model is a dummy variable whether the individual is only an active user of e-cigarettes or only an active user of conventional cigarettes. Both models also control for three groups of variables: healthy activities, demographics, and region. Healthy activities include the duration of high-impact physical activity, eating vegetables, and eating fruits. Demographics consists of age, marital status, and gender, while regional represents the main island on which the respondents live in Indonesia.

As a sensitivity analysis, we use the negative binomial regression to analyze the association between dual users and single e-cigarette users with disease multimorbidity. This choice was dictated by the nature of the dependent variable (count data), namely, the number of diseases reported by an individual. The analysis uses two models with the same primary independent variable in the logit model and also adjusts for the same control variables (health activities, demographic, and regional). In addition, we added the incidence rate ratio (IRR) to highlight the magnitude of risk for having multimorbidity as the coefficients derived from the negative binomial model could not be interpreted directly. In general, our study considers p<0.05 and p<0.01, as statistically significant.

## RESULTS

### Descriptive statistics

Table 1 shows the respondent descriptive statistics provide several demographic patterns (for an unweighted sample see Supplementary file Table 1). The respondents were primarily males (97.1%), lived in Java and Sumatra (80%), and aged on average 37.3 years. Based on their smoking behaviors, the respondents can be divided into three groups, dual users (2.71%), e-cigarette single users (0.11%), and conventional cigarette single users (97.18%). The average age of single conventional smokers was 37.6 years, which was higher than that of dual users (26.3 years) and single e-cigarette users (23.8 years). Furthermore, e-cigarette single users had the highest proportion of females (10.53%) compared to conventional cigarette single users (2.93%) and dual users (3.03%). Additionally, NCDs and activities reported by the respondents are also presented in Table 1.

**Table 1:** Respondent descriptive statistics (weighted): 2018 Indonesia Riskesdas survey, all smokers aged 15–64 years (N=58342892)

*Variable*	Category*	*All %*	*Dual user %*	*Single e-cigarette user %*	*Single conventional cigarette user %*
**User status**					
Type of user	Dual user	2.71			
	Single user	97.29			
Single e-cigarette user	True	0.11			
	False	99.89			
Single conventional cigarette user	True	97.18			
	False	2.82			
**Comorbidities**					
Asthma	Yes	1.78	2.80	5.47	1.75
	No	98.22	97.20	94.53	98.25
Diabetes	Yes	1.04	0.54	1.67	1.05
	No	98.96	99.46	98.33	98.95
Heart disease	Yes	1.09	0.75	0.44	1.10
	No	98.91	99.25	99.56	98.90
Stroke	Yes	0.48	0.25	0.00	0.48
	No	99.52	99.75	100.00	99.52
Hypertension	Yes	3.47	2.13	1.02	3.51
	No	96.53	97.87	98.98	96.49
Liver failure	Yes	0.33	0.45	0.00	0.33
	No	99.67	99.55	100.00	99.67
Rheumatism	Yes	5.57	4.01	1.26	5.62
	No	94.43	95.99	98.74	94.38
Broken teeth	Yes	48.13	49.67	43.96	48.09
	No	51.87	50.33	56.04	51.91
Mouth ulcer	Yes	7.62	11.29	11.67	7.51
	No	92.38	88.71	88.33	92.49
Gum disease	Yes	25.03	39.03	29.23	24.63
	No	74.97	60.97	70.77	75.37
**Number of disease complications**	Range (mean)	0–7 (0.14)	0–3 (0.11)	0–2 (0.10)	0–7 (0.14)
**Duration of high impact physical activities** (minutes/day)	Range (mean)	0–659 (124.10)	0–615 (73.40)	0–630 (42.69)	0–659 (125.61)
**Diet in past week**					
Vegetables	Yes	96.39	92.93	97.35	96.48
	No	3.61	7.07	2.65	3.52
Fruits	Yes	83.82	86.07	85.90	83.76
	No	16.18	13.93	14.10	16.24
**Sociodemographic characteristics**					
Age (years)	Range (mean)	15–64 (37.30)	15–64 (26.26)	15–57 (23.82)	15–64 (37.63)
Marital status	Married	69.22	33.56	21.32	70.27
	Not married	30.78	66.44	78.68	29.73
Gender	Male	97.06	96.97	89.47	97.07
	Female	2.94	3.03	10.53	2.93
**Residence**					
Java	True	58.20	67.87	63.80	57.93
	False	41.80	32.13	36.20	42.07
Sumatra	True	21.50	10.39	5.68	21.83
	False	78.50	89.61	94.32	78.17
Bali or Nusa Tenggara	True	5.08	4.51	8.39	5.09
	False	94.92	95.49	91.61	94.91
Kalimantan	True	5.65	7.04	13.14	5.60
	False	94.35	92.96	86.86	94.40
Sulawesi	True	7.02	9.03	8.80	6.96
	False	92.98	90.97	91.20	93.04
Maluku	True	1.09	0.48	0.18	1.11
	False	98.91	99.52	99.82	98.89
Papua	True	1.46	0.68	0.00	1.48
	False	98.54	99.32	100.00	98.52
**Total,** n		**58342892**	**1580802**	**66265**	**56695825**

* Scores: dual user=1, single user=0; yes=1, no=0; true=1, false=0; male=1, female=0; married=1, not married=0.

### Comparison between dual users and single users

Table 2 highlights that being a dual user is significantly associated with an increased probability of reporting an NCD compared to single users, except for heart disease (Supplementary file Table 2 for coefficients). Respondents that were dual users had a positive association with reporting to have liver failure (AOR=2.38; 95% CI: 2.32–2.44), asthma (AOR=1.65; 95% CI: 1.64–1.67), stroke (AOR=1.62; 95% CI: 1.57–1.68), diabetes (AOR=1.53; 95% CI: 1.50–1.57), hypertension (AOR=1.49; 95% CI: 1.48–1.51), and rheumatism (AOR=1.46; 95% CI: 1.44–1.48) compared to single users. Furthermore, the study found that dual users have a positive association with reporting to have gum diseases (AOR=1.74; 95% CI: 1.73–1.74), mouth ulcers (AOR=1.46; 95% CI: 1.45–1.46), and broken tooth (AOR=1.27; 95% CI: 1.26–1.27), compared to singles users. In addition, doing longer high-impact physical activities (except for rheumatism), consuming vegetables (except for stroke and heart diseases), and males were negatively associated with reporting NCDs. At the same time, age was positively associated with reporting NCDs (except for mouth ulcers and gum diseases).

**Table 2:** NCDs logit regression, dual user vs single user: 2018 Indonesia Riskesdas survey, all smokers aged 15–64 years (N=58342892)

*Variables*	*Statistics*	*Asthma*	*Diabetes*	*Heart disease*	*Hypertension*	*Stroke*	*Liver failure*	*Rheumatism*	*Broken tooth*	*Mouth ulcer*	*Gum disease*
Dual user	AOR	1.65	1.53	1.00	1.49	1.62	2.38	1.46	1.27	1.46	1.74
	SE	0.00840	0.0170	0.00955	0.00862	0.0264	0.0300	0.00615	0.00210	0.00380	0.00295
	p	**<0.001**	**<0.001**	**0.766**	**<0.001**	**<0.001**	**<0.001**	**<0.001**	**<0.001**	**<0.001**	**<0.001**
	95% CI	1.64–1.67	1.50–1.57	0.98–1.02	1.48–1.51	1.571.68	2.32–2.44	1.44–1.48	1.26–1.27	1.45–1.46	1.73–1.74
Duration of high impact physical activities (minutes/day)	AOR	0.99	0.99	0.99	0.99	0.99	1.00	1.00			
	SE	6.37e–06	1.12e–05	8.17e–06	4.85e–06	1.68e–05	1.36e–05	3.23 e–06			
	p	**<0.001**	**<0.001**	**<0.001**	**<0.001**	**<0.001**	**<0.001**	**<0.001**			
	95% CI	0.99–0.99	0.99–0.99	0.99–0.99	0.99–0.99	0.99–0.99	1.00–1.00	1.00–1.00			
Eating vegetables in the last week	AOR	0.75	0.73	1.03	0.65	1.11	0.86	0.80	0.96	0.86	0.82
	SE	0.00355	0.00477	0.00733	0.00234	0.0125	0.0106	0.00248	0.00138	0.00219	0.00131
	p	**<0.001**	**<0.001**	**<0.001**	**<0.001**	**<0.001**	**<0.001**	**<0.001**	**<0.001**	**<0.001**	**<0.001**
	95% CI	0.75–0.76	0.72–0.73	1.01–1.04	0.65–0.66	1.08–1.13	0.84–0.88	0.80–0.81	0.96–0.96	0.86–0.87	0.82–0.83
Eating fruits in the last week	AOR								0.98	1.04	0.98
	SE								0.000716	0.00144	0.000820
	p								**<0.001**	**<0.001**	**<0.001**
	95% CI								0.98–0.98	1.04–1.05	0.98 - 0.98
Age (years)	AOR	1.01	1.09	1.04	1.08	1.11	1.03	1.05	1.01	0.99	0.99
	SE	9.56e–05	0.000121	0.000116	6.98e–05	0.000191	0.000219	5.36e–05	2.48e–05	4.70e–05	2.84e–05
	p	**<0.001**	**<0.001**	**<0.001**	**<0.001**	**<0.001**	**<0.001**	**<0.001**	**<0.001**	**<0.001**	**<0.001**
	95% CI	1.01–1.01	1.09–1.09	1.04–1.04	1.07–1.08	1.11–1.11	1.03–1.03	1.05–1.05	1.01–1.01	0.99–0.99	0.99–0.99
Marital status	AOR	0.82	1.64	0.97	1.26	0.81	2.18	1.52	1.29	1.18	1.20
	SE	0.00205	0.00728	0.00321	0.00278	0.00428	0.0165	0.00268	0.000871	0.00151	0.000937
	p	**<0.001**	**<0.001**	**<0.001**	**<0.001**	**<0.001**	**<0.001**	**<0.001**	**<0.001**	**<0.001**	**<0.001**
	95% CI	0.81–0.82	1.62–1.65	0.96–0.97	1.26–1.27	0.80–0.82	2.15–2.22	1.51–1.52	1.29–1.29	1.18–1.19	1.20–1.20
Gender	AOR	0.649	0.83	0.61	0.45	1.35	0.71	0.58	0.79	0.65	0.67
	SE	0.00304	0.00466	0.00334	0.00126	0.0128	0.00786	0.00150	0.00125	0.00167	0.00114
	p	**<0.001**	**<0.001**	**<0.001**	**<0.001**	**<0.001**	**<0.001**	**<0.001**	**<0.001**	**<0.001**	**<0.001**
	95% CI	0.64–0.66	0.82–0.84	0.61–0.62	0.45–0.45	1.32–1.37	0.70–0.73	0.58–0.58	0.79–0.79	0.65–0.65	0.66–0.67
Living in Sumatera	AOR	0.77	0.94	1.05	1.04	1.11	0.89	0.98	1.03	1.09	0.85
	SE	0.00214	0.00317	0.00338	0.00192	0.00532	0.00526	0.00144	0.000686	0.00137	0.000677
	p	**<0.001**	**<0.001**	**<0.001**	**<0.001**	**<0.001**	**<0.001**	**<0.001**	**<0.001**	**<0.001**	**<0.001**
	95% CI	0.76–0.77	0.94–0.95	1.05–1.06	1.03–1.04	1.10–1.12	0.88–0.90	0.98–0.99	1.03–1.03	1.09–1.10	0.85–0.85
Living in Bali or Nusa Tenggara	AOR	1.20	0.83	0.84	0.91	0.80	0.91	0.82	0.89	1.15	1.04
	SE	0.00515	0.00557	0.00554	0.00337	0.00821	0.0100	0.00245	0.00109	0.00257	0.00146
	p	**<0.001**	**<0.001**	**<0.001**	**<0.001**	**<0.001**	**<0.001**	**<0.001**	**<0.001**	**<0.001**	**<0.001**
	95% CI	1.19–1.21	0.82–0.84	0.83–0.85	0.90–0.92	0.78–0.81	0.90–0.93	0.81–0.82	0.89–0.90	1.15–1.16	1.04–1.04
Living in Kalimantan	AOR	1.41	1.06	1.15	1.36	1.46	0.65	1.07	1.17	1.16	1.08
	SE	0.00533	0.00614	0.00622	0.00401	0.0111	0.00785	0.00268	0.00136	0.00245	0.00142
	p	**<0.001**	**<0.001**	**<0.001**	**<0.001**	**<0.001**	**<0.001**	**<0.001**	**<0.001**	**<0.001**	**<0.001**
	95% CI	1.40–1.42	1.05–1.08	1.14–1.16	1.35–1.36	1.44–1.49	0.64–0.67	1.06–1.07	1.17–1.17	1.16–1.17	1.08–1.08
Living in Sulawesi	AOR	1.14	0.97*	1.35	1.01	1.04	0.75	0.88	1.62	1.40	1.52
	SE	0.00432	0.00541	0.00625	0.00307	0.00853	0.00774	0.00215	0.00172	0.00250	0.00172
	p	**<0.001**	**<0.001**	**<0.001**	**<0.001**	**<0.001**	**<0.001**	**<0.001**	**<0.001**	**<0.001**	**<0.001**
	95% CI	1.13–1.15	0.96–0.98	1.34–1.36	1.00–1.02	1.03–1.06	0.74–0.77	0.87–0.88	1.61–1.62	1.39–1.40	1.52–1.53
Living in Maluku	AOR	0.98	0.54	1.32	0.78	1.00	1.83	0.62	1.44	1.11	1.52
	SE	0.00960	0.00953	0.0148	0.00645	0.0200	0.0301	0.00434	0.00369	0.00519	0.00410
	p	0.037	**<0.001**	**<0.001**	**<0.001**	0.986	**<0.001**	**<0.001**	**<0.001**	**<0.001**	**<0.001**
	95% CI	0.96–1.00	0.52–0.56	1.29–1.35	0.76–0.79	0.96–1.04	1.77–1.89	0.61–0.63	1.43–1.45	1.10–1.12	1.52–1.53
Living in Papua	AOR	1.51	0.75	1.07	1.08	1.21	1.19	1.98	1.19	0.67	0.95
	SE	0.0106	0.0107	0.0117	0.00704	0.0212	0.0210	0.00759	0.00263	0.00335	0.00245
	p	**<0.001**	**<0.001**	**<0.001**	**<0.001**	**<0.001**	**<0.001**	**<0.001**	**<0.001**	**<0.001**	**<0.001**
	95% CI	1.49–1.53	0.73–0.78	1.05–1.10	1.06–1.09	1.17–1.25	1.15–1.24	1.96–1.99	1.18–1.20	0.67–0.68	0.94–0.96
Constant	AOR	0.03	0.0002	0.004	0.005	4.76e–05	0.001	0.01	0.74	0.18	0.89
	SE	0.000188	3.17e–06	4.42e–05	2.55e–05	8.15e–07	1.97e–05	5.37e–05	0.00169	0.000690	0.00223
	p	**<0.001**	**<0.001**	**<0.001**	**<0.001**	**<0.001**	**<0.001**	**<0.001**	**<0.001**	**<0.001**	**<0.001**
	95% CI	0.03–0.03	0.00–0.00	0.00–0.00	0.00–0.00	0.00–0.00	0.00–0.00	0.01–0.01	0.73–0.74	0.17–0.18	0.88–0.89
**Total,** n		58342892	58342892	58342892	58342892	58342892	58342892	58342892	58342892	58342892	58342892
χ^2^		91960	787675	147116	1.749e+06	373593	44548	1.454e+06	752475	162572	709624

AOR: adjusted odds ratio. Robust standard errors, significance level: p<0.05, p**<0.01**.

### Comparison between e-cigarette single users and conventional single users

Table 3 shows that each type of cigarette single user is associated with a higher probability of reporting a different type of NCD (Supplementary file Table 3 for coefficients). Respondents that were e-cigarette single users had a positive association with reporting having diabetes (AOR=16.01; 95% CI: 14.57–17.59), asthma (AOR=3.11; 95% CI: 3.01–3.22), mouth ulcers (AOR=1.46; 95% CI: 1.42–1.49), gum diseases (AOR=1.07; 95% CI: 1.05–1.09), and broken tooth (AOR=1.04; 95% CI: 1.03–1.06), compared to conventional cigarette single users. On the other hand, e-cigarette single users had a negative association with reporting to have rheumatism (AOR=0.54; 95% CI: 0.51–0.58), heart disease (AOR=0.61; 95% CI: 0.54–0.68), and hypertension (AOR=0.84; 95% CI: 0.78–0.90), compared to conventional cigarette single users. In addition, the controls provide results similar to those of the previous section. Doing longer high-impact physical activities (except for rheumatism), consuming vegetables (except for heart diseases), and males were negatively associated with reporting NCDs. At the same time, age was positively associated with reporting NCDs (except for mouth ulcers and gum diseases).

**Table 3:** NCDs logit regression, e-cigarette single user vs conventional cigarette single user: 2018 Indonesia Riskesdas survey, single user smokers aged 15–64 years N=56762090

*Variable*	*Statistics*	*Asthma*	*Diabetes*	*Heart disease*	*Hypertension*	*Rheumatism*	*Broken tooth*	*Mouth ulcer*	*Gum disease*
E-cigarette single user	AOR	3.11	16.01	0.61	0.84	0.54	1.04	1.46	1.07
	SE	0.0540	0.770	0.0356	0.0313	0.0190	0.00808	0.0174	0.00920
	p	**<0.001**	**<0.001**	**<0.001**	**<0.001**	**<0.001**	**<0.001**	**<0.001**	**<0.001**
	95% CI	3.01–3.22	14.57–17.59	0.54–0.68	0.78–0.90	0.51–0.58	1.03–1.06	1.42–1.49	1.05–1.09
Duration of high impact physical activities (minutes/day)	AOR	0.99	0.99	0.99	0.99	1.00			
	SE	6.44e–06	1.20e–05	8.25e–06	4.89e–06	3.25e–06			
	p	**<0.001**	**<0.001**	0.348	**<0.001**	**<0.001**			
	95% CI	0.99–0.99	0.99–0.99	0.99–0.99	0.99–0.99	1.00–1.00			
Eating vegetables in the last week	AOR	0.73	0.72	0.99	0.63	0.77	0.95	0.83	0.80
	SE	0.00350	0.00514	0.00711	0.00229	0.00242	0.00141	0.00214	0.00131
	p	**<0.001**	**<0.001**	0.348	**<0.001**	**<0.001**	**<0.001**	**<0.001**	**<0.001**
	95% CI	0.72–0.73	0.71–0.73	0.98–1.01	0.63–0.64	0.77–0.78	0.95–0.96	0.82–0.83	0.80–0.81
Eating fruits in the last week	AOR						0.98	1.05	0.98
	SE						0.000727	0.00147	0.000834
	p						0	0	0
	95% CI						0.98–0.99	1.04–1.05	0.98–0.98
Age (years)	AOR	1.02	1.06	1.04	1.08	1.05	1.01	0.99	0.99
	SE	9.67e–05	0.000194	0.000117	7.08e–05	5.43e–05	2.49e–05	4.75e–05	2.87e–05
	p	**<0.001**	**<0.001**	**<0.001**	**<0.001**	**<0.001**	**<0.001**	**<0.001**	**<0.001**
	95% CI	1.02–1.02	1.06–1.06	1.04–1.04	1.08–1.08	1.05–1.05	1.01–1.01	0.99–0.99	0.99–0.99
Marital status	AOR	0.79	1.48	0.96	1.28	1.57	1.29	1.19	1.22
	SE	0.00201	0.00756	0.00324	0.00287	0.00287	0.000886	0.00155	0.000968
	p	**<0.001**	**<0.001**	**<0.001**	**<0.001**	**<0.001**	**<0.001**	**<0.001**	**<0.001**
	95% CI	0.79–0.80	1.46–1.49	0.96–0.97	1.27–1.28	1.56–1.57	1.29–1.30	1.19–1.19	1.21–1.22
Gender	AOR	0.65	0.89	0.60	0.44	0.57	0.79	0.66	0.65
	SE	0.00315	0.00526	0.00328	0.00124	0.00149	0.00127	0.00175	0.00112
	p	**<0.001**	**<0.001**	**<0.001**	**<0.001**	**<0.001**	**<0.001**	**<0.001**	**<0.001**
	95% CI	0.65–0.66	0.88–0.90	0.60–0.61	0.44–0.44	0.56–0.57	0.79–0.79	0.66–0.67	0.65–0.65
Living in Sumatera	AOR	0.77	1.01	1.05	1.04	0.99	1.03	1.09	0.85
	SE	0.00217	0.00356	0.00340	0.00193	0.00146	0.000692	0.00138	0.000684
	p	**<0.001**	**<0.001**	**<0.001**	**<0.001**	**<0.001**	**<0.001**	**<0.001**	**<0.001**
	95% CI	0.77–0.78	1.01–1.02	1.05–1.06	1.03–1.04	0.99–0.99	1.03–1.03	1.09–1.09	0.85–0.85
Living in Bali or Nusa Tenggara	AOR	1.22	0.90	0.86	0.92	0.81	0.90	1.17	1.05
	SE	0.00531	0.00629	0.00566	0.00343	0.00247	0.00111	0.00265	0.00149
	p	**<0.001**	**<0.001**	**<0.001**	**<0.001**	**<0.001**	**<0.001**	**<0.001**	**<0.001**
	95% CI	1.21–1.23	0.89–0.91	0.85–0.87	0.91–0.93	0.81–0.82	0.90–0.90	1.17–1.18	1.05–1.06
Living in Kalimantan	AOR	1.44	1.01	1.14	1.37	1.08	1.17	1.18	1.08
	SE	0.00554	0.00642	0.00627	0.00409	0.00273	0.00138	0.00253	0.00145
	p	**<0.001**	**0.0309**	**<0.001**	**<0.001**	**<0.001**	**<0.001**	**<0.001**	**<0.001**
	95% CI	1.43–1.45	1.00–1.03	1.13–1.15	1.36–1.38	1.07–1.08	1.16–1.17	1.17–1.18	1.07–1.08
Living in Sulawesi	AOR	1.16	0.95	1.37	1.01	0.88	1.62	1.41	1.53
	SE	0.00448	0.00575	0.00639	0.00310	0.00218	0.00176	0.00258	0.00176
	p	**<0.001**	**<0.001**	**<0.001**	**0.00344**	**<0.001**	**<0.001**	**<0.001**	**<0.001**
	95% CI	1.15–1.17	0.94–0.96	1.36–1.38	1.00–1.01	0.87–0.88	1.62–1.62	1.40–1.41	1.53–1.53
Living in Maluku	AOR	0.99	0.56	1.34	0.79	0.62	1.46	1.11	1.52
	SE	0.00982	0.0106	0.0149	0.00654	0.00439	0.00375	0.00526	0.00413
	p	0.670	**<0.001**	**<0.001**	**<0.001**	**<0.001**	**<0.001**	**<0.001**	**<0.001**
	95% CI	0.98–1.01	0.54–0.58	1.31–1.36	0.77–0.80	0.61–0.63	1.45–1.47	1.10–1.12	1.51–1.53
Living in Papua	AOR	1.55	0.68	1.07	1.06	1.97	1.19	0.68	0.95
	SE	0.0109	0.0111	0.0117	0.00700	0.00762	0.00264	0.00340	0.00248
	p	**<0.001**	**<0.001**	**<0.001**	**<0.001**	**<0.001**	**<0.001**	**<0.001**	**<0.001**
	95% CI	1.53–1.57	0.66–0.71	1.05–1.09	1.04–1.07	1.95–1.98	1.18–1.19	0.67–0.69	0.95–0.96
Constant	AOR	0.03	0.001	0.005	0.005	0.01	0.74	0.18	0.92
	SE	0.000193	1.93e–05	4.73e–05	2.63e–05	5.50e–05	0.00172	0.000708	0.00235
	p	**<0.001**	**<0.001**	**<0.001**	**<0.001**	**<0.001**	**<0.001**	**<0.001**	**<0.001**
	95% CI	0.02–0.03	0.00–0.00	0.00–0.00	0.00–0.00	0.01–0.01	0.73–0.74	0.17–0.18	0.92–0.93
**Total**, n		56762090	56762090	56762090	56762090	56762090	56762090	56762090	56762090
χ^2^		88645	179350	141954	1.722e+06	1.429e+06	741547	129199	553053

AOR: adjusted odds ratio. Robust standard errors, significance level: p<0.05, p**<0.01**.

### Multimorbidity analysis

Lastly, the study utilizes the negative binomial method as an additional analysis to find the association between dual users and disease multimorbidity. First, our study revealed that dual users have greater multimorbidity than single users (Supplementary file Table 4). Using the IRR, the study found that the incidence rate of multimorbidity among dual users was 1.5 times higher than for single users. Second, when only comparing single users, our study indicates that single e-cigarette users have more multimorbidity compared to conventional cigarette single users (Supplementary file Table 5). Using the IRR, the study found that the incidence rate of multimorbidity among e-cigarette single users was 1.5 times higher than for conventional cigarette single users.

## DISCUSSION

Our study highlights the dangers of using e-cigarettes which contain several toxicants that increase the risk to specific NCDs. A study has shown a significant association between e-cigarette usage and asthma^13,14^. E-cigarette usage also causes health issues in the mouth, gums, and mouth ulcers^15-17^. There is a greater susceptibility of e-cigarette consumers to develop changes in oral biological tissue as manifested by plaque indices, peri-implant bone loss, worse radiographic bone rates, and higher levels of proinflammatory cytokines than conventional cigarette users^15^.

The first finding of the study shows that dual usage of e-cigarettes and conventional cigarettes has a positive association with reporting NCDs, which is aligned with several studies. Dual users had an increased probability of reporting NCDs, including heart disease, hypertension, stroke, and asthma^8,18^. Dual users have higher cardiovascular risks than single users of conventional cigarettes and non-smokers^8,19^. The components of heart disease, including increased waist circumference, elevated triglycerides, and low high-density lipoprotein (HDL)-cholesterol, were more prevalent in dual users than in never smokers^8^. Furthermore, dual users are also associated with higher cardiopulmonary health risks and chronic obstructive pulmonary disease than cigarette single users^20,21^. The additive effect of dual users was found to be related with greater nicotine dependence and urinary cotinine compared to conventional cigarette single users and non-smokers^8,22^. Moreover, another study had shown that there were significantly higher levels of toxicants, such as benzene, ethylene oxide, acrylonitrile, acrolein, and acrylamide in the urine of adolescent dual users than in adolescent e-cigarette single users^23^.

The second part of the study compared the risk of reporting NCDs between e-cigarette single users and conventional cigarette single users, in which the current literature provides mixed results. While e-cigarettes contain toxicants that harm health, it is important to consider the magnitude of potential harm when compared to conventional cigarettes. A study had shown that e-cigarette single users were associated with lower volatile organic compounds exposure compared to conventional cigarette single users^24^. Furthermore, another study had shown e-cigarette and conventional cigarette users to have immune-related gene suppression in the nasal area, where e-cigarette users had a higher level of suppression^25^. Furthermore, e-cigarettes contained heavy metals (nickel and chromium) that are not found in conventional cigarettes^26^. On the other hand, Bozier et al.^27^ and Marques et al.^28^ showed through a systematic review that exclusive e-cigarette usage provides less harm than exclusive conventional cigarette usage. Conventional cigarette users who converted to e-cigarettes were found to have improved oral health^29,30^. Conventional cigarette single users and dual users, but not e-cigarette single users, had a higher risk of cardiovascular diseases compared with non-smokers^19^. Furthermore, former smokers who converted to e-cigarettes had lower odds of respiratory outcomes compared to conventional cigarette single users^31^. This is due to the toxicants in e-cigarettes being found to be on a lower dose compared to conventional cigarettes, thus leading to less severe symptoms^28^.

The second finding in our study provides some alignment and contrast with the established literature. Regarding cardiovascular related diseases, the study shows that conventional cigarette single users have a higher likelihood to report heart diseases and hypertension compared to e-cigarette single users. Compared to conventional cigarette single users, e-cigarette single users were found to have a lower increase in blood pressure^32^. However, the study has also shown e-cigarette single users had a higher likelihood of reporting asthma and mouth diseases, which is not in line with other studies^27,31^. This difference may be due to the analysis of other studies focusing on subjects that had converted to e-cigarettes. In contrast, our study focused on current e-cigarette single users (not factoring in previous smoking status). Furthermore, there is a significant difference in the subject sample size compared to the current study. These findings indicate the need for future studies that can provide long-term effects of e-cigarette usage.

### Strengths and limitations

This study’s strength is that the secondary dataset used in the study, Riskesdas, is a nationally representative survey enhanced using sample weights for an Indonesia case study. The study also demonstrates that the epidemiological transition due to NCDs, which is more common in the elderly group, can shift to a younger age due to the behavior of dual users (electronic and conventional cigarettes). However, this study is limited because the measurement period for acquiring the disease is limited to one year (cross-sectional data). In contrast, the incidence of disease due to smoking tends to be chronic, or the manifestation of disease develops over a relatively long period. This finding is specific for Indonesia. Further research is still needed using cohort analysis to assess the consistency of this study’s results and the frequency of using e-cigarettes, including liquid refills. However, this study’s finding of an association of NCDs reported by the participant in the short-term, proves that dual user and e-cigarette behavior should be avoided. Another limitation of the study is the issue of recall bias during the data collection of chronic disease and smoking behavior. As the respondents provide information on past diagnosis and smoking behavior, the study can be subject to recall bias. Recall bias may cause underestimation or overestimation of the actual effect or association^33^. However, the Riskesdas does a pilot survey to check the validity of the questionnaire answers to reduce recall bias issues^33^. Furthermore, in regard to smoking behavior, the survey asks the respondent about smoking behavior in the last month. Limiting the timeframe of recall also reduces the recall bias^33^. Regardless, the issue of recall bias may persist. The study also acknowledges that NCDs have multiple risk factors, such as the case of diabetes; however, this study only focuses on the risk caused by smoking behavior.

## CONCLUSIONS

E-cigarette consumption is not a substitute for conventional cigarettes since most e-cigarette users are dual users of conventional cigarettes. This happens because both are products that contain nicotine as an addictive substance. This study found that dual users were positively associated with reporting to have diseases and had higher disease multimorbidity than single users. Moreover, when comparing single users of e-cigarettes and conventional cigarettes, each type of product was associated with a higher likelihood of reporting having a different NCD. Single e-cigarette users were more likely to report having asthma, diabetes, mouth diseases, and disease multimorbidity than conventional cigarette single users. On the other hand, single, conventional cigarette users were associated with a higher likelihood of reporting to have hypertension, rheumatic and heart diseases than single e-cigarette users.

Therefore, it is suggested that quitting smoking is better than switching to other products. We recommend enhancing and enforcing policies that control e-cigarette and conventional cigarette consumption simultaneously. This is because the primary reason for the use of e-cigarettes is the perception that e-cigarettes pose less risk than conventional cigarettes. However, this perception is incorrect. E-liquid and e-cigarette use doses can be one of the causes of e-cigarette users having a greater risk of having NCDs compared to conventional cigarette users. Moreover, due to the complementary nature of e-cigarettes, controlling the consumption of conventional cigarettes will also affect the consumption of e-cigarettes.

## Data Availability

The data supporting this research cannot be made available for privacy or other reasons.
