# Supplementary material for: Relieving or aggravating the burden: Non-communicable diseases of dual users of electronic and conventional cigarette in Indonesia
Source: Tob Induc Dis. 2024 Jan 10;22:10.18332/tid/175755. doi: 10.18332/tid/175755 (PMC10777476; doi:10.18332/tid/175755)
Supplement: Supplementary file 1 [file TID-22-05-s1.pdf]

Appendix

Appendix 1: Respondent Descriptive Statistics (Unweighted): 2018 Indonesia Riskesdas Survey, All 15–64-Year-Old Smokers (n = 174,917)

Variable	Category	Statistics			
		All Sample	Dual User	Single E-Cigarette User	Single Conventional Cigarette User
Respondent is Dual User (1 = Dual User; 0 = Single User)	Dual User (%)	2.08			
	Single User (%)	97.92			
Respondent is Single E-Cigarette User (1 = True; 0 = False)	True (%)	0.09			
	False (%)	99.91			
Respondent is Single Conventional Cigarette User (1 = True; 0 = False)	True (%)	97.84			
	False (%)	2.16			
Has Been Diagnosed Having Asthma (1 = Yes; 0 = No)	Yes (%)	1.81	2.70	3.90	1.79
	No (%)	98.19	97.30	96.10	98.21
Has Been Diagnosed Having Diabetes (1 = Yes; 0 = No)	Yes (%)	1.10	0.61	1.30	1.11
	No (%)	98.90	99.39	98.70	98.89
Has Been Diagnosed Having Heart Disease (1 = Yes; 0 = No)	Yes (%)	1.17	0.85	0.65	1.18
	No (%)	98.83	99.15	99.35	98.82
Has Been Diagnosed Having Stroke (1 = Yes; 0 = No)	Yes (%)	0.52	0.41	0.00	0.53
	No (%)	99.48	99.59	100.00	99.47
Has Been Diagnosed Having Hypertension (1 = Yes; 0 = No)	Yes (%)	3.86	2.29	0.65	3.89
	No (%)	96.14	97.71	99.35	96.11
Has Been Diagnosed Having Liver Failure (1 = Yes; 0 = No)	Yes (%)	0.35	0.36	0.00	0.35
	No (%)	99.65	99.64	100.00	99.65
Has Been Diagnosed Having Rheumatism (1 = Yes; 0 = No)	Yes (%)	6.13	3.66	1.30	6.19
	No (%)	93.87	96.34	98.70	93.81
Has Been Diagnosed Having Broken Teeth (1 = Yes; 0 = No)	Yes (%)	49.74	50.66	40.26	49.73
	No (%)	50.26	49.34	59.74	50.27
Has Been Diagnosed Having Mouth Ulcer (1 = Yes; 0 = No)	Yes (%)	7.76	11.37	9.74	7.68
	No (%)	92.24	88.63	90.26	92.32
Has Been Diagnosed Having Gum Disease (1 = Yes; 0 = No)	Yes (%)	24.94	39.65	30.52	24.62
	No (%)	75.06	60.35	69.48	75.38
Number of Disease Complications (Number of Diseases)	Min-Max (Mean)	0 - 7 (0.15)	0 - 3 (0.11)	0 - 2 (0.08)	0 - 7 (0.15)
Duration of High Impact Physical Activities (Minutes/Day)	Min-Max (Mean)	0 - 659 (138.23)	0 - 615 (82.04)	0 - 630 (43.77)	0 - 659 (139.51)
Eating Vegetables in the Last Week (1 = Yes; 0 = No)	Yes (%)	96.75	93.42	96.75	96.83
	No (%)	3.25	6.58	3.25	3.17
Eating Fruits in the Last Week (1 = Yes; 0 = No)	Yes (%)	82.24	85.27	89.61	82.17
	No (%)	17.76	14.73	10.39	17.83
Respondent Age (Years)	Min-Max (Mean)	15 - 64 (38.78)	15 - 64 (26.98)	15 - 57 (24.38)	15 - 64 (39.04)
Marital Status (1 = Married; 0 = Not Married)	Married (%)	72.98	35.77	25.97	73.81
	Not Married (%)	27.02	64.23	74.03	26.19
Gender (1 = Male; 0 = Female)	Male (%)	96.33	96.78	90.91	96.33
	Female (%)	3.67	3.22	9.09	3.67
Living in Java (1 = True; 0 = False)	True (%)	32.90	45.32	44.16	32.62
	False (%)	67.10	54.68	55.84	67.38
Living in Sumatra (1 = True; 0 = False)	True (%)	31.26	17.40	9.74	31.58
	False (%)	68.74	82.60	90.26	68.42
Living in Bali or Nusa Tenggara (1 = True; 0 = False)	True (%)	8.02	6.94	13.64	8.04
	False (%)	91.98	93.06	86.36	91.96
Living in Kalimantan (1 = True; 0 = False)	True (%)	9.17	12.14	20.78	9.09
	False (%)	90.83	87.86	79.22	90.91

Living in Sulawesi (1 = True; 0 = False)	True (%)	13.21	15.58	11.04	13.16
	False (%)	86.79	84.42	88.96	86.84
Living in Maluku (1 = True; 0 = False)	True (%)	2.52	1.16	0.65	2.55
	False (%)	97.48	98.84	99.35	97.45
Living in Papua (1 = True; 0 = False)	True (%)	2.92	1.46	0.00	2.95
	False (%)	97.08	98.54	100.00	97.05
Observations		174,917	3,632	154	171,131

Appendix 2: NCDs Regression, Dual User Vs Single User (Coefficients): 2018 Indonesia Riskesdas Survey, All 15–64-Year-Old Smokers (n = 58,342,892)

	Logit	Logit	Logit	Logit	Logit	Logit	Logit	Logit	Logit	Logit
	Asthma	Hypertension	Stroke	Liver Failure	Rheumatism	Diabetes	Heart Disease	Broken Tooth	Mouth Ulcer	Gum Diseases
VARIABLE	Coef	Coef	Coef	Coef	Coef	Coef	Coef	Coef	Coef	Coef
Dual User (1 = Dual-User; 0 = Single User)	0.502**	0.402**	0.484**	0.867**	0.375**	0.426**	0.00283	0.237**	0.376**	0.551**
	(0.00508)	(0.00577)	(0.0163)	(0.0126)	(0.00423)	(0.0111)	(0.00952)	(0.00165)	(0.00261)	(0.00170)
Duration of High Impact Physical Activities (Minutes/Day)	-0.000645**	-0.00107**	-0.00295**	-0.000269**	0.000411**	-0.00282**	-0.000795**			
	(6.37e-06)	(4.86e-06)	(1.69e-05)	(1.36e-05)	(3.23e-06)	(1.13e-05)	(8.18e-06)			
Eating Vegetables in the Last Week (1 = Yes; 0 = No)	-0.282**	-0.429**	0.101**	-0.149**	-0.223**	-0.321**	0.0256**	-0.0402**	-0.148**	-0.193**
	(0.00470)	(0.00360)	(0.0113)	(0.0123)	(0.00310)	(0.00657)	(0.00715)	(0.00144)	(0.00254)	(0.00159)
Eating Fruits in the Last Week (1 = Yes; 0 = No)								-0.0189**	0.0430**	-0.0237**
								(0.000730)	(0.00138)	(0.000840)
Age (Years)	0.0140**	0.0727**	0.103**	0.0251**	0.0477**	0.0892**	0.0355**	0.00758**	-0.0115**	-0.0145**
	(9.43e-05)	(6.49e-05)	(0.000173)	(0.000214)	(5.11e-05)	(0.000111)	(0.000112)	(2.46e-05)	(4.75e-05)	(2.88e-05)
Marital Status (1 = Married; 0 = Not Married)	-0.201**	0.233**	-0.211**	0.781**	0.416**	0.493**	-0.0334**	0.253**	0.169**	0.182**
	(0.00250)	(0.00221)	(0.00529)	(0.00754)	(0.00177)	(0.00445)	(0.00332)	(0.000676)	(0.00127)	(0.000781)
Gender (1 = Male; 0 = Female)	-0.432**	-0.802**	0.298**	-0.337**	-0.545**	-0.188**	-0.488**	-0.238**	-0.430**	-0.405**
	(0.00469)	(0.00280)	(0.00950)	(0.0110)	(0.00260)	(0.00562)	(0.00543)	(0.00158)	(0.00257)	(0.00171)
Living in Sumatera (1 = True; 0 = False)	-0.263**	0.0341**	0.102**	-0.120**	-0.0162**	-0.0583**	0.0530**	0.0294**	0.0899**	-0.160**
	(0.00278)	(0.00185)	(0.00480)	(0.00594)	(0.00147)	(0.00336)	(0.00321)	(0.000666)	(0.00125)	(0.000794)
Living in Bali or Nusa Tenggara (1 = True; 0 = False)	0.182**	-0.0944**	-0.227**	-0.0957**	-0.199**	-0.184**	-0.173**	-0.113**	0.142**	0.0385**
	(0.00429)	(0.00371)	(0.0103)	(0.0110)	(0.00299)	(0.00670)	(0.00659)	(0.00123)	(0.00223)	(0.00140)
Living in Kalimantan (1 = True; 0 = False)	0.345**	0.304**	0.380**	-0.425**	0.0655**	0.0609**	0.138**	0.157**	0.150**	0.0767**
	(0.00378)	(0.00296)	(0.00762)	(0.0120)	(0.00251)	(0.00577)	(0.00542)	(0.00117)	(0.00211)	(0.00132)
Living in Sulawesi (1 = True; 0 = False)	0.129**	0.0102**	0.0427**	-0.282**	-0.130**	-0.0334**	0.299**	0.479**	0.334**	0.420**
	(0.00379)	(0.00303)	(0.00817)	(0.0103)	(0.00245)	(0.00559)	(0.00464)	(0.00107)	(0.00179)	(0.00113)
Living in Maluku (1 = True; 0 = False)	-0.0204*	-0.254**	-0.000340	0.604**	-0.483**	-0.611**	0.279**	0.366**	0.104**	0.421**
	(0.00980)	(0.00832)	(0.0200)	(0.0164)	(0.00704)	(0.0176)	(0.0112)	(0.00256)	(0.00468)	(0.00269)
Living in Papua (1 = True; 0 = False)	0.412**	0.0742**	0.189**	0.177**	0.680**	-0.282**	0.0697**	0.174**	-0.394**	-0.0490**
	(0.00705)	(0.00654)	(0.0176)	(0.0176)	(0.00384)	(0.0142)	(0.0109)	(0.00221)	(0.00497)	(0.00257)
Constant	-3.664**	-5.367**	-9.952**	-6.813**	-4.437**	-8.159**	-5.417**	-0.304**	-1.739**	-0.119**
	(0.00734)	(0.00547)	(0.0171)	(0.0179)	(0.00454)	(0.0111)	(0.00995)	(0.00228)	(0.00393)	(0.00251)
Observations	58,342,892	58,342,892	58,342,892	58,342,892	58,342,892	58,342,892	58,342,892	58,342,892	58,342,892	58,342,892
chi2	91960	1.749e+06	373593	44548	1.454e+06	787675	147116	752475	162572	709624

Note: Coef = Coefficient, Robust standard errors in parentheses, significance level (alpha): * (5%) and ** (1%)

Appendix 3: NCDs E-Cigarette Single User Vs Conventional Cigarette Single User (Coefficients): 2018 Indonesia Riskesdas Survey, 15–64-Year-Old Single User Smokers (n = 56,762,090)

	Logit	Logit	Logit	Logit	Logit	Logit	Logit	Logit
	Asthma	Hypertension	Rheumatism	Diabetes	Heart Diseases	Broken Tooth	Mouth Ulcer	Gum Diseases
VARIABLE	Coef	Coef	Coef	Coef	Coef	Coef	Coef	Coef
E-Cigarette Single User (1 = E-Cigarette Single User; 0 = Conventional Cigarette Single User)	1.136**	-0.174**	-0.612**	2.016**	-0.500**	0.0405**	0.375**	0.0671**
	(0.0173)	(0.0372)	(0.0350)	(0.0306)	(0.0588)	(0.00776)	(0.0120)	(0.00860)
Duration of High Impact Physical Activities (Minutes/Day)	-0.000600**	-0.00108**	0.000408**	-0.00277**	-0.000812**			
	(6.45e-06)	(4.89e-06)	(3.25e-06)	(1.13e-05)	(8.26e-06)			
Eating Vegetables in the Last Week (1 = Yes; 0 = No)	-0.318**	-0.459**	-0.256**	-0.324**	-0.00672	-0.0488**	-0.191**	-0.219**
	(0.00482)	(0.00362)	(0.00312)	(0.00665)	(0.00716)	(0.00148)	(0.00259)	(0.00163)
Eating Fruits in the Last Week (1 = Yes; 0 = No)						-0.0166**	0.0436**	-0.0225**
						(0.000739)	(0.00141)	(0.000853)
Age (Years)	0.0147**	0.0730**	0.0478**	0.0892**	0.0351**	0.00761**	-0.0112**	-0.0145**
	(9.53e-05)	(6.58e-05)	(5.17e-05)	(0.000112)	(0.000113)	(2.47e-05)	(4.80e-05)	(2.91e-05)
Marital Status (1 = Married; 0 = Not Married)	-0.230**	0.245**	0.450**	0.471**	-0.0363**	0.257**	0.175**	0.196**
	(0.00253)	(0.00225)	(0.00183)	(0.00445)	(0.00336)	(0.000685)	(0.00130)	(0.000796)
Gender (1 = Male; 0 = Female)	-0.421**	-0.817**	-0.562**	-0.194**	-0.507**	-0.236**	-0.412**	-0.428**
	(0.00480)	(0.00282)	(0.00261)	(0.00563)	(0.00545)	(0.00160)	(0.00264)	(0.00173)
Living in Sumatera (1 = True; 0 = False)	-0.259**	0.0372**	-0.0119**	-0.0479**	0.0510**	0.0302**	0.0869**	-0.160**
	(0.00282)	(0.00186)	(0.00148)	(0.00337)	(0.00323)	(0.000672)	(0.00127)	(0.000803)
Living in Bali or Nusa Tenggara (1 = True; 0 = False)	0.197**	-0.0841**	-0.206**	-0.166**	-0.153**	-0.109**	0.157**	0.0516**
	(0.00436)	(0.00373)	(0.00303)	(0.00670)	(0.00660)	(0.00124)	(0.00226)	(0.00142)
Living in Kalimantan (1 = True; 0 = False)	0.365**	0.314**	0.0748**	0.0614**	0.129**	0.155**	0.164**	0.0746**
	(0.00385)	(0.00298)	(0.00253)	(0.00582)	(0.00551)	(0.00119)	(0.00215)	(0.00135)
Living in Sulawesi (1 = True; 0 = False)	0.146**	0.00898**	-0.132**	-0.0328**	0.313**	0.482**	0.342**	0.426**
	(0.00388)	(0.00307)	(0.00249)	(0.00564)	(0.00467)	(0.00109)	(0.00183)	(0.00115)
Living in Maluku (1 = True; 0 = False)	-0.00420	-0.242**	-0.474**	-0.598**	0.289**	0.377**	0.107**	0.418**
	(0.00986)	(0.00833)	(0.00706)	(0.0176)	(0.0112)	(0.00258)	(0.00472)	(0.00272)
Living in Papua (1 = True; 0 = False)	0.436**	0.0545**	0.675**	-0.269**	0.0671**	0.172**	-0.387**	-0.0467**
	(0.00708)	(0.00663)	(0.00388)	(0.0142)	(0.0109)	(0.00223)	(0.00501)	(0.00259)
Constant	-3.661**	-5.349**	-4.424**	-8.136**	-5.350**	-0.304**	-1.734**	-0.0814**
	(0.00751)	(0.00553)	(0.00459)	(0.0112)	(0.00996)	(0.00233)	(0.00401)	(0.00255)
Observations	56,762,090	56,762,090	56,762,090	56,762,090	56,762,090	56,762,090	56,762,090	56,762,090
chi2	88645	1.722e+06	1.429e+06	760642	141954	741547	129199	553053

Note: Coef = Coefficient, Robust standard errors in parentheses, significance level (alpha): * (5%) and ** (1%)

Appendix 4: Complication Regression, Dual User Vs. Single User: 2018 Indonesia Riskesdas Survey, All 15–64-Year-Old Smokers (n = 58,342,892)

	Negative Binomial	
	Complication	
VARIABLES	Coefficient	IRR
Dual User (1 = Dual-User; 0 = Single User)	0.420**	1.522**
	(0.003)	(0.00390)
Duration of High Impact Physical Activities (Minutes/Day)	-0.000**	1.000**
	(0.000)	(2.30e-06)
Eating Vegetables in the Last Week (1 = Yes; 0 = No)	-0.244**	0.783**
	(0.002)	(0.00151)
Age (Years)	0.050**	1.051**
	(0.000)	(3.46e-05)
Marital Status (1 = Married; 0 = Not Married)	0.172**	1.187**
	(0.001)	(0.00128)
Gender (1 = Male; 0 = Female)	-0.505**	0.604**
	(0.002)	(0.000952)
Living in Sumatera (1 = True; 0 = False)	-0.033**	0.968**
	(0.001)	(0.000931)
Living in Bali or Nusa Tenggara (1 = True; 0 = False)	-0.105**	0.901**
	(0.002)	(0.00168)
Living in Kalimantan (1 = True; 0 = False)	0.165**	1.180**
	(0.002)	(0.00186)
Living in Sulawesi (1 = True; 0 = False)	-0.011**	0.989**
	(0.002)	(0.00155)
Living in Maluku (1 = True; 0 = False)	-0.214**	0.807**
	(0.004)	(0.00341)
Living in Papua (1 = True; 0 = False)	0.382**	1.465**
	(0.003)	(0.00407)
lnalpha	-0.504**	0.604**
	(0.003)	(0.00157)
Constant	-3.425**	0.0326**
	(0.003)	(9.32e-05)
Observations	58,342,892	
chi2	3.200e+06	

Note: Robust standard errors are in parentheses, significance level (alpha): * (5%) and ** (1%)

Appendix 5: Complication Regression E-Cigarette Single User Vs. Conventional Cigarette Single User): 2018 Indonesia Riskesdas Survey, 15–64-Year-Old Single User Smokers (n = 56,762,090)

	Negative Binomial	
	Complication	
**VARIABLES**	Coefficient	IRR
E-Cigarette Single User (1 = E-Cigarette Single User; 0 = Conventional Cigarette Single User)	0.430**	1.538**
	(0.0119)	(0.0183)
Duration of High Impact Physical Activities (Minutes/Day)	−0.000440**	1.000**
	(2.32e-06)	(2.32e-06)
Eating Vegetables in the Lathan= Yes; 0 = No)	−0.270**	0.764**
	(0.00195)	(0.00149)
Age (Years)	0.0499**	1.051**
	(3.33e-05)	(3.50e-05)
Marital Status (1 = Married; 0 = Not Married)	0.179**	1.196**
	(0.00110)	(0.00131)
Gender than0 = Female)	−0.517**	0.596**
	(0.00158)	(0.000945)
Living in Sumatera (1 = True; 0 = False)	−0.0281**	0.972**
	(0.000968)	(0.000942)
Living in Bali or Nusa Tenggara (1 = True; 0 = False)	−0.101**	0.904**
	(0.00189)	(0.00171)
Living in Kalimantan (1 = True; 0 = False)	0.174**	1.190**
	(0.00160)	(0.00190)
Living in Sulawesi (1 = True; 0 = False)	−0.00834**	0.992**
	(0.00159)	(0.00158)
Living in Maluku (1 = True; 0 = False)	−0.203**	0.817**
	(0.00423)	(0.00346)
Living in Papua (1 = True; 0 = False)	0.381**	1.464**
	(0.00281)	(0.00411)
lnalpha	−0.502**	0.606**
	(0.00262)	(0.00159)
Constant	−3.407**	0.0332**
	(0.00290)	(9.60e-05)
Observations	56,762,090	
chi2	3.123e+06	

Note: Robust standard errors are in parentheses, significance level (alpha): * (5%) and ** (1%)

© 2024 Moeis F.R. et al.
